# A robust gene regulatory network inference method base on Kalman filter and linear regression

**DOI:** 10.1371/journal.pone.0200094

**Published:** 2018-07-12

**Authors:** Jamshid Pirgazi, Ali Reza Khanteymoori

**Affiliations:** Department of Computer Engineering, Engineering Faculty, University of Zanjan, Zanjan, Iran; Instituto Nacional de Medicina Genomica, MEXICO

## Abstract

The reconstruction of the topology of gene regulatory networks (GRNs) using high throughput genomic data such as microarray gene expression data is an important problem in systems biology. The main challenge in gene expression data is the high number of genes and low number of samples; also the data are often impregnated with noise. In this paper, in dealing with the noisy data, Kalman filter based method that has the ability to use prior knowledge on learning the network was used. In the proposed method namely (*KFLR*), in the first phase by using mutual information, the noisy regulations with low correlations were removed. The proposed method utilized a new closed form solution to compute the posterior probabilities of the edges from regulators to the target gene within a hybrid framework of Bayesian model averaging and linear regression methods. In order to show the efficiency, the proposed method was compared with several well know methods. The results of the evaluation indicate that the inference accuracy was improved by the proposed method which also demonstrated better regulatory relations with the noisy data.

## Introduction

The study of gene regulatory networks (GRNs) structure is important in understanding cellular function. GRNs are typically represented by graphs in which the nodes represent the genes and the edges show the regulatory or interaction between genes. There are many methods for inference of GRNS. One of these methods is computational methods. Many computational methods have been proposed in the literature to model GRNs. These methods can be classified into the co-expression based methods [1], supervised learning-based methods [2,3], model-based methods [4,5] and information theory-based methods [6,7]. Co-expression based methods have low complexity but lack inference direction of interaction. The supervised learning methods such as GENIES [8] and SIRENE [9] require information about some interactions in order to learn the models.

Model-based methods can be categorized into ordinary differential equation [10], multiple linear regression [11], Boolean networks [12] and probabilistic graphical models including Bayesian Network (BN) and Dynamic Bayesian Network (DBN) [13]. They infer GRNs with high accuracy and can identify direction of interaction. However, these methods are time consuming and require many parameters to be set up, and thus cannot be used for large-scale networks. There are two suggestions for addressing this problem: Searching the optimal graph from all possible graphs and using decomposition technique in regression based methods for network structure inference. The inference of regulatory interactions for *N* genes is decomposed into *N* independent sub-problems, with sub-problems inferring the regulators of a target gene. Narimani et all proposed a new Bayesian network reverse engineering method using ordinary differential equations with the ability to include non-linearity. In this method, Expectation Propagation is used for approximate Bayesian inference [14]. Due to Bayesian network (BN) methods cannot handle large-scale networks in [15] present a novel method, namely local Bayesian network (LBN), to infer GRNs from gene expression data by using the network decomposition strategy and false-positive edge elimination scheme.

There are many significant advantages to use Bayesian network model. Bayesian networks can be easily understood and allow researchers to use their domain expert knowledge for determine the Bayesian network structure. When sample size is small Bayesian networks are less influenced and they use the probability theory, which is suitable for dealing with noise in biological data. Furthermore, Bayesian networks where complete data are not available, can produce relatively accurate prediction. Although a few disadvantages exist, such as computational complexity and need to set many parameters, therefore they cannot be used for large-scale networks.

To address this problem within this context, this paper presents a new method that uses Bayesian model averaging based on Kalman filter and linear regression to infer GRNs. In this method, a new solution is applied to calculate the posterior probabilities of the edges from possible regulators to the target gene, which leads to high prediction accuracy and high computational efficiency. This method is the best performer among well-known existing methods in the *DREAM4* in silico challenge and *IRMA* Dataset [16–17].

Another important category of GRN inference methods is based on regression methods, which are used to predict one target gene based on one or more input genes such as artificial neural networks (ENFRN) [18], support vector machines (SIRENE)], rotation forest (GENIRF) [19], random forests (GENIE3) [20] and Bayesian Model Averaging for Linear Regression (BMALR) [21].

Furthermore, information theory-based methods are used for inferencing GNRs, such as conditional mutual information (*CMI*) [6] and mutual information (*MI*) [15]. This method can be used for large scale networks.

MI measures the dependency between two genes. A higher mutual information value for two genes shows that one gene is related with the other. However, MI cannot distinguish indirect regulators from direct ones. Consequently, this leads to possible false positives [22]. Although CMI-based methods are able to distinguish indirect regulators from direct ones, they cannot locate the directions of interactions in the network and also in some cases underestimate the interactions strength. These network inference methods such as Context Likelihood of Relatedness (CLR) [23], Weighted Gene Co-Expression Network Analysis (WGCNA) [24], Algorithm for the Reconstruction of Accurate Cellular Network’s (ARACNE) [25], Relevance Networks (RN) [26] and Minimum-Redundancy–Maximum-Relevance Network (MRNET) [27] assume that correlation between genes expression are indicative of a regulatory interaction. In [28] for capture coarse-grained dynamics propose a new mutual information based Boolean network inference (MIBNI) method. In This method, using mutual information first selected a set of initial regulatory genes, and then by iteratively swapping a pair of genes between the selected regulatory genes and the other genes, improves the dynamics prediction accuracy.

The rest of this paper is organized as follows. Details of the Kalman filter are given in section two. Conditional Mutual Information is given in section three. The proposed method is presented in section four. In section five, the results of the proposed method are shown on data collection *DREAM4* and other datasets. Finally, conclusions are summarized in section six.

## Kalman filter

To infer gene regulatory network, one way is to find the Bayesian network structure. This is normally achieved by maximizing the likelihood of the observed dataset (maximum likelihood) or the posterior probability of the structure given the observed data (maximum a posteriori). In this paper, because the data are time series and contain noise, the Kalman filter is used to find the Bayesian network structure [29].

**Fig 1:**
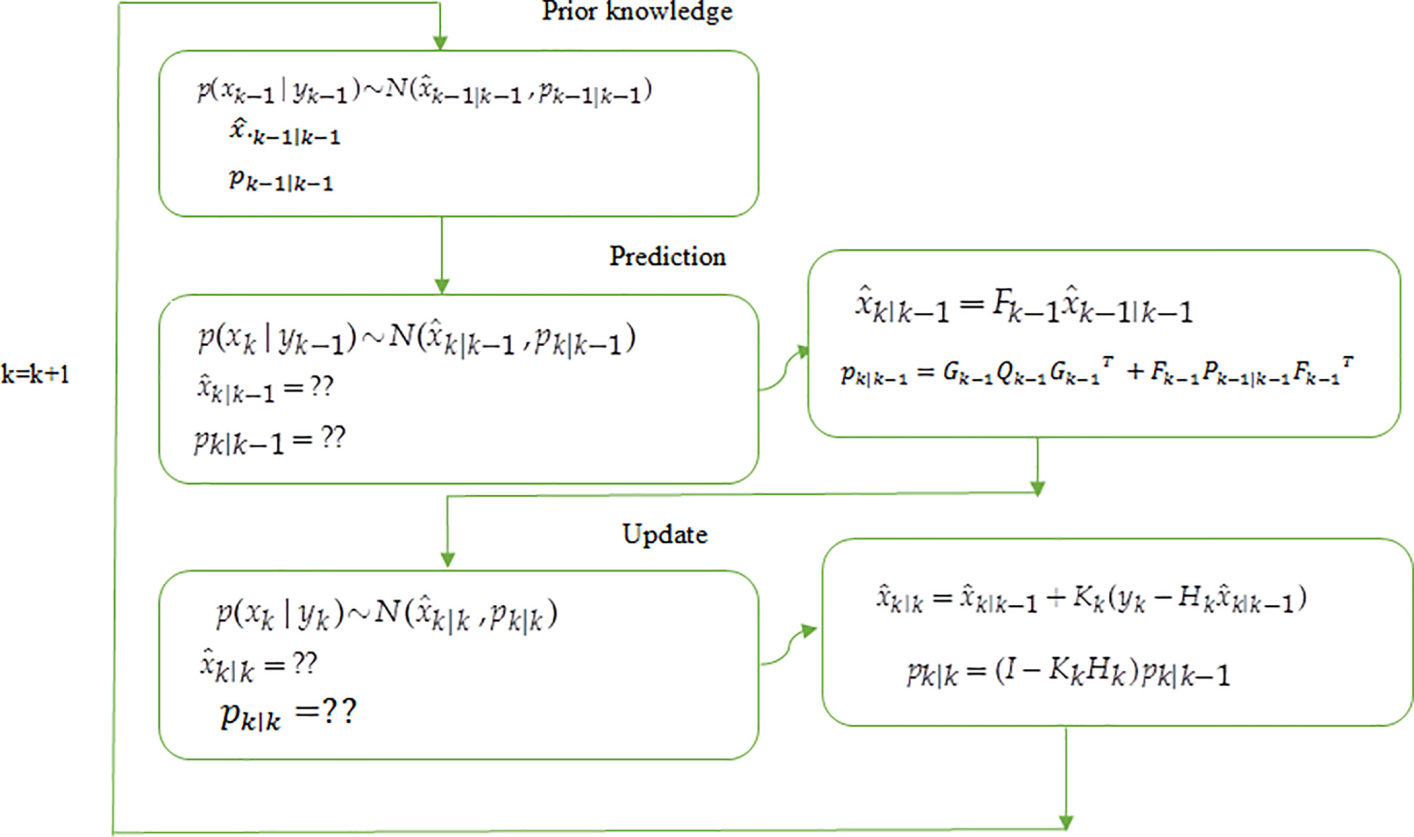
Kalman filter phases in the proposed method.

Kalman filter is an algorithm that uses a series of measurements observed over time, containing statistical noise. Applying the Kalman filter for this purpose, assuming input and output, is given as:

$$x_{k+1}=F_{k}x_{k}+G_{k}w_{k}$$

$$y_{k}=H_{k}x_{k}+D_{k}v_{k}$$ (1)

where *F*_*k*_ is the state transition model which is applied to the previous state *x*_*k*_, *G*_*k*_ is the control matrix which is applied to the *w*_*k*_. *w*_*k*_ is the process noise which is assumed to be drawn from a zero mean multivariate normal distribution with covariance *Q*_*k*_. *H*_*k*_ is the observation model which maps the true state space into the observed space, *D*_*k*_ is the control matrix which is applied to the *v*_*k*_, and *v*_*k*_ is the observation noise which is assumed to be zero mean Gaussian white noise with covariance *R*_*k*_ [29]. The details of Kalman filter is shown in Fig 1. First, prior probability *p*(*x*_*k*−1_|*y*_*k*−1_) has a random value and is related to prior knowledge as follows:

$$p(x_{k-1}|y_{k-1})∼N({\hat{x}}_{k-1|k-1},p_{k-1|k-1})$$ (2)

Prediction and updating phases can alternatively be used to calculate the posterior probability. In the prediction phase, the value of *p*(*x*_*k*_|*y*_*k*−1_) is obtained as follows:

$$p(x_{k}|y_{k-1})∼N({\hat{x}}_{k|k-1},p_{k|k-1})$$ (3)

Instead of predicting this probability, the mean and variance, *x*_*k*|*k*−1_ and *p*_*k*|*k*−1_, are predicted respectively. In the updating phase, the value of *p*(*x*_*k*_|*y*_*k*_) is obtained as follows. As a matter of, in this step the parameters of the mean and variance of the posterior probability are updated.

$$p(x_{k}|y_{k})∼N({\hat{x}}_{k|k},p_{k|k})$$ (4)

This probability is achieved over time and periodically until the posterior probability is calculated. By using the following equations, the mean and variance can be obtained as follows:

$${\hat{x}}_{k|k-1}=F_{k-1}{\hat{x}}_{k-1|k-1}$$ (5)

$$p_{k|k-1}=G_{k-1}Q_{k-1}G_{k-1}{}^{T}+F_{k-1}P_{k-1|k-1}F_{k-1}{}^{T}$$ (6)

$${\hat{x}}_{k|k}={\hat{x}}_{k|k-1}+K_{k}(y_{k}-H_{k}{\hat{x}}_{k|k-1})$$ (7)

$$p_{k|k}=(I-K_{k}H_{k})p_{k|k-1}$$ (8)

Where *K*_*k*_ is Kalman rate and it is calculated as follows:

$$K_{k}=p_{k|k-1}H_{k}^{T}{\left(H_{k}p_{k|k-1}H_{k}^{T}+D_{k}R_{k}D_{k}^{T}\right)}^{-1}$$ (9)

## Conditional mutual information

Mutual Information (*MI*) and Conditional Mutual Information (*CMI*) have been used to construct GRNs [30] owing to their ability to detect nonlinear dependencies between genes with Gaussian noise.

The mutual information (*MI*) is a measure of the mutual dependence between two genes *X*_*i*_ and *X*_*j*_. Thus, its value can be used to evaluate the strengths between genes. For measuring the conditional dependency between two genes *X*_*i*_ and *X*_*j*_ given another gene *X*_*k*_, *CMI* can be used, which can quantify the undirected regulation. For discrete variables *X* and *Y*, *MI* is defined as [31]:

$$MI(X,Y)=-\sum_{x\in X,y\in Y}p(x,y)\log\frac{p(x,y)}{p(x)p(y)}=H(X)+H(Y)-H(X,Y)$$ (10)

where *p(x)* and *p(y)* are the marginal probability distributions of *X* and *Y*, respectively, *p (x*, *y)* is the joint probability distribution of *X* and *Y*, *H*(*X*,*Y*) is the joint entropy of *X* and *Y*, and *H(X)* and *H(Y)* are the entropies of *X* and *Y*, respectively. *CMI* between two variables *X* and *Y* given variable *Z* is defined as [31]:

$$CMI(X,Y|Z)=-\sum_{x\in X,y\in Y,z\in Z}p(x,y,z)\log\frac{p(x,y|z)}{p(x|z)p(y|z)}=H(X,Z)+H(Y,Z)-H(X,Y,Z)$$ (11)

where *H*(*X*,*Z*), *H*(*Y*,*Z*) and *H*(*X*,*Y*,*Z*) are the joint entropies, and *p*(*x*,*y*|*z*), *p*(*x*|*z*) and *p*(*y*|*z*) are the conditional probability distributions, respectively.

## Proposed method

**Fig 2:**
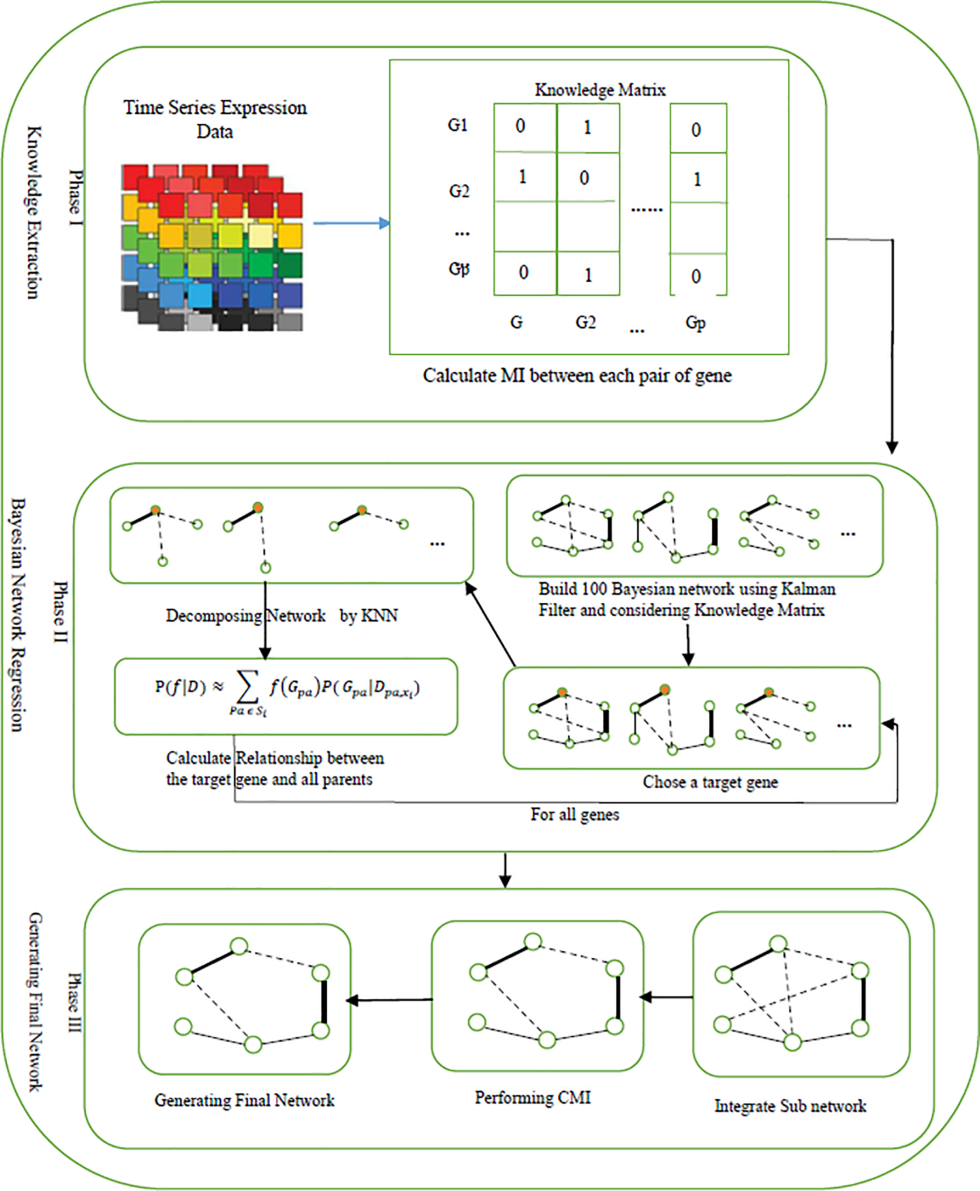
Schematic diagram of proposed method.

*KFLR* constructs GRNs using Bayesian network and linear regression which lead to a directed graph of regulatory interactions between genes with high accuracy. This method mainly consists of three distinct phases. In the first phase prior knowledge is extracted from the data using *MI*, then in the next phase Bayesian network is constructed based on prior knowledge and Kalman filter, and in the last phase the network is modified using *CMI*. The proposed method is described in Fig 2. In the next subsection, detailed description of each of these phases will be presented.

### Phase 1: Knowledge extraction with *MI*

In this step, the *MI* values between all genes are computed and the knowledge matrix is created. If *MI (i*, *j)* is smaller than a threshold, the cell (*i*,*j*) in the knowledge matrix will be zero, otherwise this cell is one.

#### Phase 2: Building Bayesian network using Kalman filter

For inferring gene regulatory network, the proposed idea is based on prior knowledge from the knowledge matrix, and Kalman filter is used to construct Bayesian network. The proposed method integrates a Bayesian model averaging method with a linear regression approach. A new method is used to calculate the posterior probability edges based on Kalman filter. In the proposed method, linear regression is used on the target gene and all combinations of other genes. The final score of the edge between the parent and target genes, is the sum of the all posterior probability of the linear regression models containing this edge.

### Bayesian model averaging

One methods for inference of gene regulatory network is finding the structure *N* of Bayesian network that better explains the data. There are many methods for finding Bayesian network structure such as maximizing the likelihood of the observed data (maximum likelihood, ML) or the posterior probability of the structure *N* given the observed data (maximum posteriori, MAP). This paper makes use of Kalman filter in achieving the posterior probability. There exist a lot of Bayesian network structures that best describe the data when the number of observations in gene data are limited. We can find best structure using heuristic search.

But the heuristic search methods have high computational complexity and do not guarantee global optimal. Thus, Bayesian model averaging can be used instead of searching for the best structure among the existing Bayesian structures. In other words, the probability of an edge (*f*) given the observed dataset (*D*) between node *i* and *j* in a structure (*N*) can be calculated with the posterior probability of *f*:

$$P(f|D)=\sum_{N}f\left(N\right)P(N|D)$$ (12)

This probability shows the posterior probability of *f* given the observed dataset (*D*). In this equation, if the Bayesian network *N* contains edge *f*, *f (N)* equals to *1*, otherwise it is *0*. Therefore, 100 Bayesian networks are built with different structures using Kalman filter and then the final score edge from node *i* to node *j*, can be obtained based on Bayesian structures posterior probability of this edge. In other words, in the construction of Bayesian networks using Kalman filter theory, each node *X*_*i*_, has a probability distribution *P*(*X*_*i*_|*Parents*(*X*_*i*_)), that shows the effect of parent nodes on this node to be numerical. In this step, the parent sets which are obtained with prior knowledge of the first phase, are checked and genes having smaller *MI* degree than a threshold are not selected with gene *X*_*i*_. In order to have an accurate estimate for posterior probability of the edge, with k-nearest neighbor (*kNN*) method, the network is decomposed into a set of smaller sub networks according to the relationship among genes in the network. In the graph structure, according to their shortest path distance the *k* nearest neighbors of each gene are selected. In this paper, the *k*-nearest neighbors with *k = 2* containing the Markova blanket of the gene are applied for each gene in order to decompose a global network to a set of sub networks.

$$X_{i}=\sum_{i\neq j}w_{ji}X_{j}+e_{i}$$ (13)

where *X*_*i*_ is the expression level of gene *i*, *w*_*ji*_ is a weight between gene *i* and *j* and showing the effect of gene *j* on gene *i*. If *w*_*ji*_ is zero, then in the gene regulatory network there is no edge from *j* to *i*. If *w*_*ji*_ is non-zero, *j* is one of the *i*’s candidate regulators (parents) and *ε*_*i*_ denotes the noise. The posterior probability for each edge calculate base on the sum of the posterior probabilities of all the sub structures containing the edge [19]. Using the following equation, the posterior probability of an edge feature *f* is calculated:

$$P\left(f|D\right)\approx \sum_{Pa\in S_{i}}f\left(N_{Pa}\right)P(N_{Pa}|D_{Pa,x_{i}})$$ (14)

where *S*_*i*_ is the set of all possible parent sets of *X*_*i*_. *X*_*i*_ is the target of the edge feature *f*. $D_{Pa,x_{i}}$ denotes the data restricted to *X*_*i*_ and the genes in *Pa*. *N*_*Pa*_ is a sub structure that is composed of the edges from the genes in *Pa*, a parent set of gene *X*_*i*_. If the sub structure *N*_*Pa*_ contains *f*, *f*(*N*_*Pa*_) is equal to *1*, otherwise it is *0*.

### Phase 3: Modifying the network

After gene regulatory network inference, the network is modified to achieve better results. *MI* method commonly cannot estimate the regulation degrees between genes. Because it does not consider the joint regulations into two or more genes, the rate of false positive edges is high. In this phase, by computing the first-order *CMI (i*, *j|k)* and second-order *CMI (i*, *j|k*, *l)*, false positive edges are removed. By so doing, if *CMI (i*, *j|k)* (or *CMI (i*, *j|k*, *l)*) is smaller than a threshold *α*, the edge between genes *i* and *j* is removed from the network.

## Experimental result

### Data set

The *DREAM* (for ‘‘Dialogue for Reverse Engineering Assessments and Methods”) initiative organizes an annual reverse engineering competition called the *DREAM* challenge [27]. The goal of the *DREAM4* In silico network challenge is to reverse engineer gene regulation networks from simulated steady state and time series data. There are three sub-challenges consists of five networks called in silico Size 10, In silico Size 100, and In silico Size 100 Multifactorial. In the time series data, for networks of size 10, there is 5 different time series, for networks of size 100, there is 10 different time series. Each time series has 21 time points [28]. All networks and data were generated with Gene Net Weaver (*GNW*) version 2.0 [32]. Network topologies were obtained by extracting sub networks from transcriptional regulatory networks of E. coli and S. cerevisiae (see S1 Data).

Another dataset we have used is the IRM dataset. *IRMA* network is a subnetwork embedded in Saccharomyces cerevisiae which consist of 5 genes: *CBF1*, *GAL4*, *SWI5*, *GAL80*, and *ASH1*. Gene expression data are time-series and include switch-off data and switch-on data. The switch-off data is taken from 4 experiments and the switch-on data is taken from 5 experiments, with a total of 142 samples measured (see S1 Data) [33].

### Performance metrics

The proposed method is evaluated using the area under the precision versus recall curve and receiver operating characteristic (*ROC*) curve for the whole set of link predictions for a network.

A precision-recall (*PR*) curve plots fraction of retrieved instances that are relevant (Precision) versus the fraction of relevant instances that are retrieved (*Recall*), whereas a *ROC* curve plots the true positive rate versus the false positive rate [34]. To summarize these curves, the *DREAM* organizers proposed different statistics. *AUPR and AUROC are* respectively the area under the *PR* and *ROC* curve. *AUPR p-value and AUROC p-value are* the probability that random ordering of the potential links is given or larger of *AUPR* and *AUROC*.

The overall *p*-values: p_aupr_ and p_auroc_ of the five networks constituting each *DREAM4* sub challenge were defined as the geometric mean of the individual *p*-values, as shown in Eq 15 [35]:

$$\bar{p}=\sqrt[{5}]{p_{1}\cdot p_{2}\cdot p_{3}\cdot p_{4}\cdot p_{5}}$$ (15)

The overall score for each method was the log-transformed geometric mean of the overall *AUROC p*-value and the overall *AUPR p*-value, as shown in Eq 16 [35]:

$$Overall=-\frac{1}{2}\cdot log_{10}({\bar{P}}_{aupr}\cdot {\bar{P}}_{auroc})$$ (16)

### Performance comparison on the DREAM4 dataset

In this section, evaluation of five inferred sub network using the proposed method before and after adding noise into data has been studied and to demonstrate the performance, the proposed method has been compared with thirteen common methods in the field of gene regulatory networks construction. Methods used for comparison are as follows:

***GENIE3***, is an algorithm for inferring regulatory networks from expression data using tree-based methods. The implementation of matlab codes by its authors and with default parameters and protocols are used [18]. ***BMALR*** is an algorithm for inferring cellular regulatory networks with Bayesian model averaging for linear regression algorithm. The author’s system code is used [19]. ***CLR*** [21], ***ARACNE*** [23] and ***MRNET*** [25] algorithms: These three algorithms have been implemented by the minet package into R Language. ***BGRMI***, Bayesian Gene Regulation Model Inference, a model-based method for inferring GRNs from time-course gene expression data. BGRMI uses a Bayesian framework to calculate the probability of different models of GRNs and a heuristic search strategy to scan the model space efficiently [36]. ***G1DBN*** is a method based on dynamic Bayesian network [37]. ***NARROMI*** is a noise and redundancy reduction technique improves accuracy of gene regulatory network inference [5]. ***TIGRESS*,** this method solves the network inference problem by using a feature selection technique (LARS) combined with stability selection. In the method Web-based platform is performed [38]. ***GENIRF***, this method decomposes the prediction of a gene regulatory network between p genes into p different regression problems. Each regression problem is constructed with singular value decomposition and rotation forest [17]. ***MIBNI***, in this method, first selected a set of initial regulatory genes using mutual information, and then, improves the dynamics prediction accuracy by iteratively swapping a pair of genes between the selected regulatory genes and the other genes [26]. The implementation of java codes by its authors and with default parameters and protocols are used. ***FBISC***, in this method, expectation propagation is used for approximate Bayesian inference [14]. The implementation of C# codes by its authors and with default parameters and protocols are used. ***CMI2NI*,** *CMI2* is used to quantify the mutual information between two genes given a third one through calculating the Kullback–Leibler divergence between the postulated distributions of including and excluding the edge between the two genes [6]. The implementation of matlab codes by its authors and with default parameters and protocols are used.

In the following, the results in the form of *AUPR* and *AUROC* values, *ROC* and *PR* curves are examined and an overall score is calculated for each method. As earlier mentioned, 5 sub networks in *DREAM4* dataset were used for evaluation. The goal of each 5 sub network is finding the rank for edges and directional regulatory relations. Table 1 shows the *AUPR* and *AUROC* values for different methods in 5 sub networks without noise. Comparing stability against noise, Table 2 shows *AUPR* and *AUROC* values for different methods in noisy data. It should be noted that 10% Gaussian noise with mean = 0 and standard deviation = 1 was added to the data. From the results, the proposed method is robust against noise than the other methods. The results show that the proposed method has higher accuracy, because of the use of the knowledge extraction phase in network constructions and removal of many false positives edges. Also, the use of Kalman filter probability theory can thus deal with noise data in which the Kalman filter removes noisy regulations.

**Table 1:** AUPR and AUROC values of common GRN methods without noise.

*Method*	*NET1*		*NET2*		*NET3*		*NET4*		*NET5*	
	*AUPR*	*AUROC*	*AUPR*	*AUROC*	*AUPR*	*AUROC*	*AUPR*	*AUROC*	*AUPR*	*AUROC*
*BMALR*	0.173	0.745	0.155	0.722	0.201	0.745	0.186	0.768	0.198	*0.758*
*GINIE3*	0.228	0.789	0.096	0.614	0.230	0.775	0.157	0.721	0.168	*0.712*
*MRNET*	0.143	0.584	0.075	0.579	0.124	0.683	0.128	0.708	0.095	*0.611*
*ARACNE*	0.165	0.634	0.108	0.611	0.174	0.679	0.143	0.709	0.154	*0.621*
*BGRMI*	**0.245**	0.804	0.118	0.71	0.185	0.696	0.213	0.784	0.154	*0.643*
*CLR*	0.179	0.782	0.109	0.635	0.238	0.787	0.154	0.712	0.163	*0.705*
*G1DBN*	0.089	0.589	0.055	0.612	0.155	0.678	0.153	0.705	0.117	*0.631*
*NARROMI*	0.122	0.713	0.105	0.665	0.192	0.706	0.167	0.713	0.186	*0.727*
*TIGRESS*	0.157	0.738	0.144	0.68	0.172	0.759	0.199	0.764	0.198	*0.747*
*GENIRF*	0.174	0.763	0.156	0.731	0.212	0.763	0.191	0.772	0.202	*0.781*
*MIBNI*	0.162	0.637	0.126	0.711	0.182	0.683	0.173	0.742	0.173	*0.725*
*FBISC*	0.167	0.635	0.173	0.598	**0.263**	0.65	0.228	0.664	0.206	*0.685*
*CMI2NI*	0.057	0.737	0.048	0.616	0.102	0.69	0.063	0.657	0.066	*0.691*
***KFLR***	0.194	**0.812**	**0.195**	**0.823**	0.235	**0.803**	**0.236**	**0.813**	**0.221**	*0.797*

**Table 2:** AUPR and AUROC values of common GRN methods with noise.

*Method*	*NET1*		*NET2*		*NET3*		*NET4*		*NET5*	
	*AUPR*	*AUROC*	*AUPR*	*AUROC*	*AUPR*	*AUROC*	*AUPR*	*AUROC*	*AUPR*	*AUROC*
*BMALR*	0.155	0.721	0.125	0.689	0.185	0.724	0.162	0.692	0.173	*0.678*
*GINIE3*	0.192	0.718	0.058	0.537	0.201	0.788	0.135	0.642	0.143	*0.612*
*MRNET*	0.065	0.582	0.072	0.573	0.108	0.589	0.11	0.645	0.098	*0.598*
*ARACNE*	0.142	0.602	0.089	0.601	0.122	0.621	0.123	0.656	0.133	*0.623*
*BGRMI*	**0.208**	0.785	0.102	0.636	0.154	0.633	0.196	0.721	0.123	*0.578*
*CLR*	0.139	0.724	0.065	0.578	0.183	0.714	0.121	0.672	0.132	*0.678*
*G1DBN*	0.054	0.521	0.043	0.578	0.12	0.602	0.118	0.654	0.092	*0.586*
*NARROMI*	0.102	0.703	0.087	0.68	0.182	0.688	0.159	0.696	0.172	*0.709*
*TIGRESS*	0.146	0.722	0.132	0.671	0.163	0.741	0.187	0.748	0.186	*0.736*
*GENIRF*	0.162	0.712	0.136	0.682	0.189	0.743	0.173	0.711	0.168	*0.691*
*MIBNI*	0.143	0.609	0.094	0.682	0.157	0.609	0.153	0.692	0.146	*0.674*
*FBISC*	0.154	0.612	0.161	0.502	0.263	0.613	0.215	0.609	0.189	*0.621*
*CMI2NI*	0.042	0.702	0.044	0.583	0.094	0.598	0.061	0.611	0.061	*0.626*
***KFLR***	0.189	**0.81**	**0.193**	**0.821**	**0.232**	**0.795**	**0.232**	**0.807**	**0.213**	*0.772*

According to Tables 1 and 2, the rate of improvement of the *KFLR* in sub network 1 is also less, while the rate of improvement is higher in sub network 2,3, 4 and 5, because of extracting more false positive edges. Therefore, with the more accurate obtained knowledge in the first phase, *KFLR* results to better network. In fact, in the Bayesian network construction phase using the Kalman filter, each node *X*_*i*_ have one conditional probability distribution *P*(*X*_*i*_|*Parents*(*X*_*i*_)) which shows the effect of parents on this node numerically. In this phase, parents are selected with obtained knowledge from first phase and not allowed to select genes which are very similar to each other. So this work changes the value of relationships between one gene and its parents in comparison with inferred network by other algorithm. When the number of extracted knowledge increases, more improvement is achieved compared with other algorithm. In *KFLR*, the refining network using *CMI* coefficient is done. This phase will improve the amounts of regulatory relations between pairs of genes using biology significant relationships between them and this work improves the results of each sub network slightly.

Table 3 shows *p*-values of *AUROC* and *AUPR* for different methods and each subnet separately. This shows that the predictions of this method is significantly better than a random guess compared to other methods. The overall scores of each method in the whole network are shown in Table 4. The results indicate that the proposed method performs better than the other methods.

**Table 3:** AUPR and AUROC p-values for DREAM4 challenge.

*Method*	*NET1*		*NET2*		*NET3*		*NET4*		*NET5*	
	*P-AUPR*	*P- AUROC*	*P-AUPR*	*P- AUROC*	*P-AUPR*	*P- AUROC*	*P-AUPR*	*P- AUROC*	*P-AUPR*	*P- AUROC*
*BMALR*	3.20E-28	3.30E-15	3.10E-34	2.10E-22	3.52E-47	8.40E-32	4.21E-41	4.36E-30	3.20E-43	*3.67E-33*
*GINIE3*	3.40E-36	3.20E-19	8.40E-21	2.10E-16	2.76E-54	8.70E-34	2.73E-34	5.42E-28	7.41E-37	*3.79E-29*
*MRNET*	2.31E-11	1.98E-09	6.23E-22	6.11E-19	4.54E-33	4.21E-22	3.46E-30	5.02E-25	2.73E-28	*9.93E-19*
*ARACNE*	6.32E-21	4.11E-20	1.25E-22	1.23E-20	5.03E-37	4.05E-25	5.99E-32	8.15E-27	5.31E-37	*7.22E-28*
*BGRMI*	4.10E-37	2.50E-21	6.33E-28	5.43E-21	5.23E-39	5.86E-25	4.51E-48	6.31E-34	4.46E-33	*4.52E-20*
*CLR*	4.50E-31	3.20E-18	2.32E-24	4.52E-18	5.72E-55	6.85E-36	3.11E-31	4.26E-27	3.72E-36	*5.31E-28*
*G1DBN*	8.24E-10	8.23E-06	1.35E-15	2.23E-15	3.43E-36	1.10E-25	1.27E-31	4.32E-26	7.02E-27	*8.76E-18*
*NARROMI*	9.13E-20	5.42E-18	1.76E-23	4.09E-20	1.32E-40	7.63E-28	2.21E-37	5.72E-27	3.25E-40	*4.81E-31*
*TIGRESS*	4.30E-22	1.27E-20	7.18E-32	3.56E-20	3.86E-38	1.65E-32	4.20E-43	3.28E-29	7.26E-42	*5.68E-33*
*GENIRF*	3.31E-29	3.51E-18	5.41E-35	2.32E-23	2.60E-48	4.27E-31	3.17E-42	4.82E-32	2.62E-44	*2.46E-32*
*MIBNI*	6.51E-23	3.19E-22	3.18E-27	4.31E-21	4.27E-41	2.82E-28	2.21E-36	3.17E-28	1.93E-37	*3.37E-30*
*FBISC*	1.41E-27	1.60E-17	6.31E-36	4.12E-19	2.43E-37	4.22E-21	1.81E-32	6.32E-23	4.81E-36	*2.17E-27*
*CMI2NI*	1.28E-10	1.58E-17	2.61E-08	3.62E-09	5.09E-22	8.09E-18	2.44E-11	2.21E-12	2.55E-12	*3.08E-16*
***KFLR***	1.63E-33	1.21E-27	7.43E-46	4.12E-32	4.43E-58	3.22E-39	2.81E-53	7.63E-35	3.61E-51	*1.35E-37*

**Table 4:** Score of common GRN methods and our method for DREAM4.

*Method*	*NET1*	*NET2*	*NET3*	*NET4*	*NET5*	*Total Score*
*BMALR*	2.10E+01	2.76E+01	3.88E+01	3.49E+01	3.75E+01	*1.60E+02*
*GINIE3*	2.70E+01	1.79E+01	4.33E+01	3.04E+01	3.23E+01	*1.51E+02*
*MRNET*	9.67E+00	1.97E+01	2.69E+01	2.69E+01	2.28E+01	*1.06E+02*
*ARACNE*	1.98E+01	2.09E+01	3.03E+01	2.87E+01	3.17E+01	*1.31E+02*
*BGRMI*	2.85E+01	2.37E+01	3.13E+01	4.03E+01	2.58E+01	*1.50E+02*
*CLR*	2.39E+01	2.05E+01	4.47E+01	2.84E+01	3.14E+01	*1.49E+02*
*G1DBN*	7.08E+00	1.48E+01	3.02E+01	2.81E+01	2.16E+01	*1.02E+02*
*NARROMI*	1.82E+01	2.11E+01	3.35E+01	3.14E+01	3.49E+01	*1.39E+02*
*TIGRESS*	2.06E+01	2.53E+01	3.46E+01	3.54E+01	3.67E+01	*1.53E+02*
*GENIRF*	2.30E+01	2.85E+01	3.90E+01	3.64E+01	3.76E+01	*1.64E+02*
*MIBNI*	2.18E+01	2.34E+01	3.40E+01	3.16E+01	3.31E+01	*1.44E+02*
*FBISC*	2.18E+01	2.68E+01	2.85E+01	2.70E+01	3.10E+01	*1.35E+02*
*CMI2NI*	1.33E+01	8.01E+00	1.92E+01	1.11E+01	1.36E+01	*6.52E+01*
***KFLR***	2.99E+01	3.83E+01	4.79E+01	4.33E+01	4.37E+01	*2.03E+02*

Recall and precision are the ratios of the numbers of correctly inferred interactions vs all interactions in the gold standard networks and the reconstructed networks respectively The Area under the PR curve (*AUPR*) provides an unbiased scalar estimate of the accuracies of the reconstructed GRNs. *ROC* curves (*AUROC*) is a measure of the overall performance of a model.

**Fig 3:**
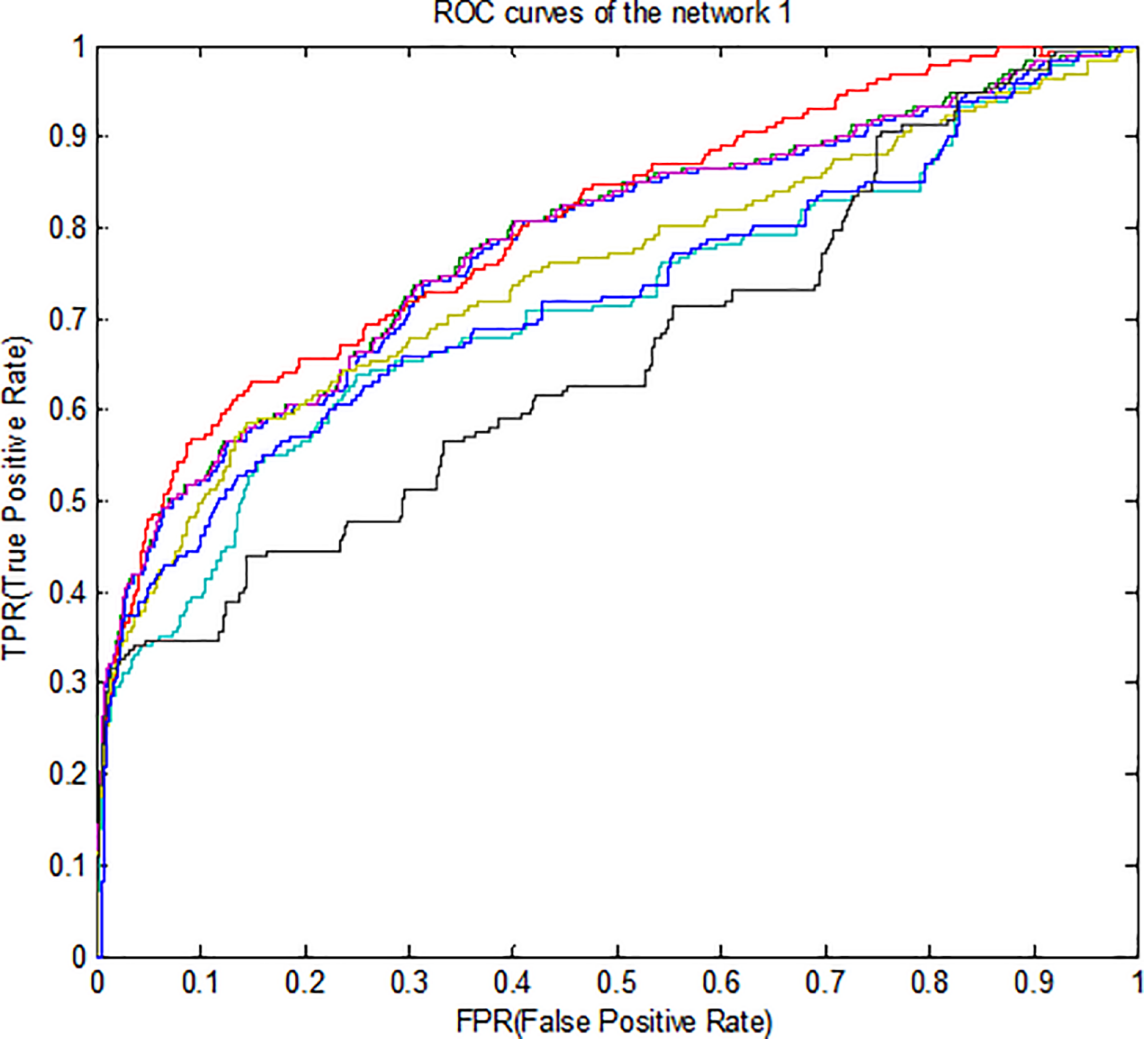
ROC curves for different methods in sub network1.

**Fig 4:**
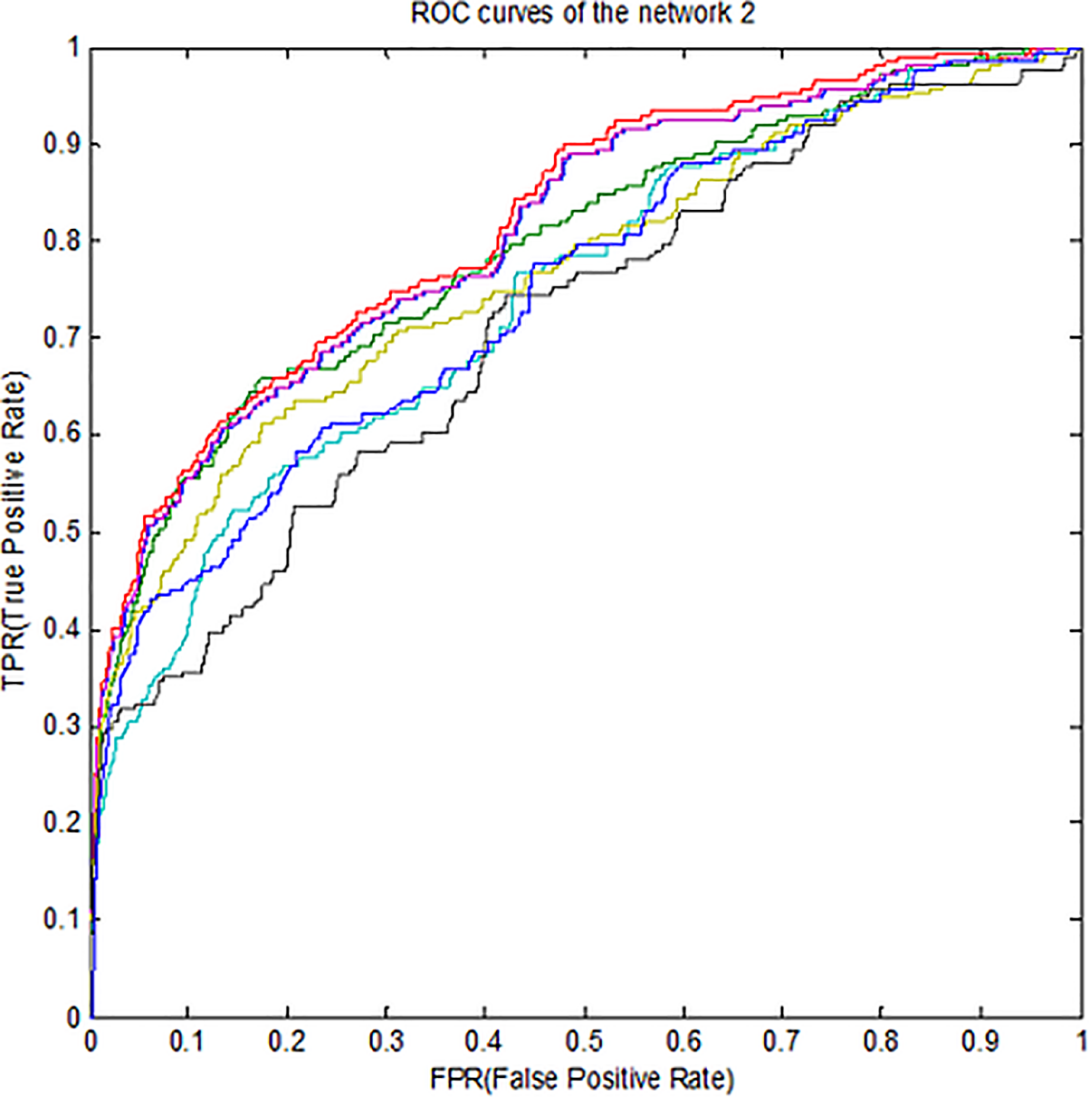
ROC curves for different methods in sub network2.

**Fig 5:**
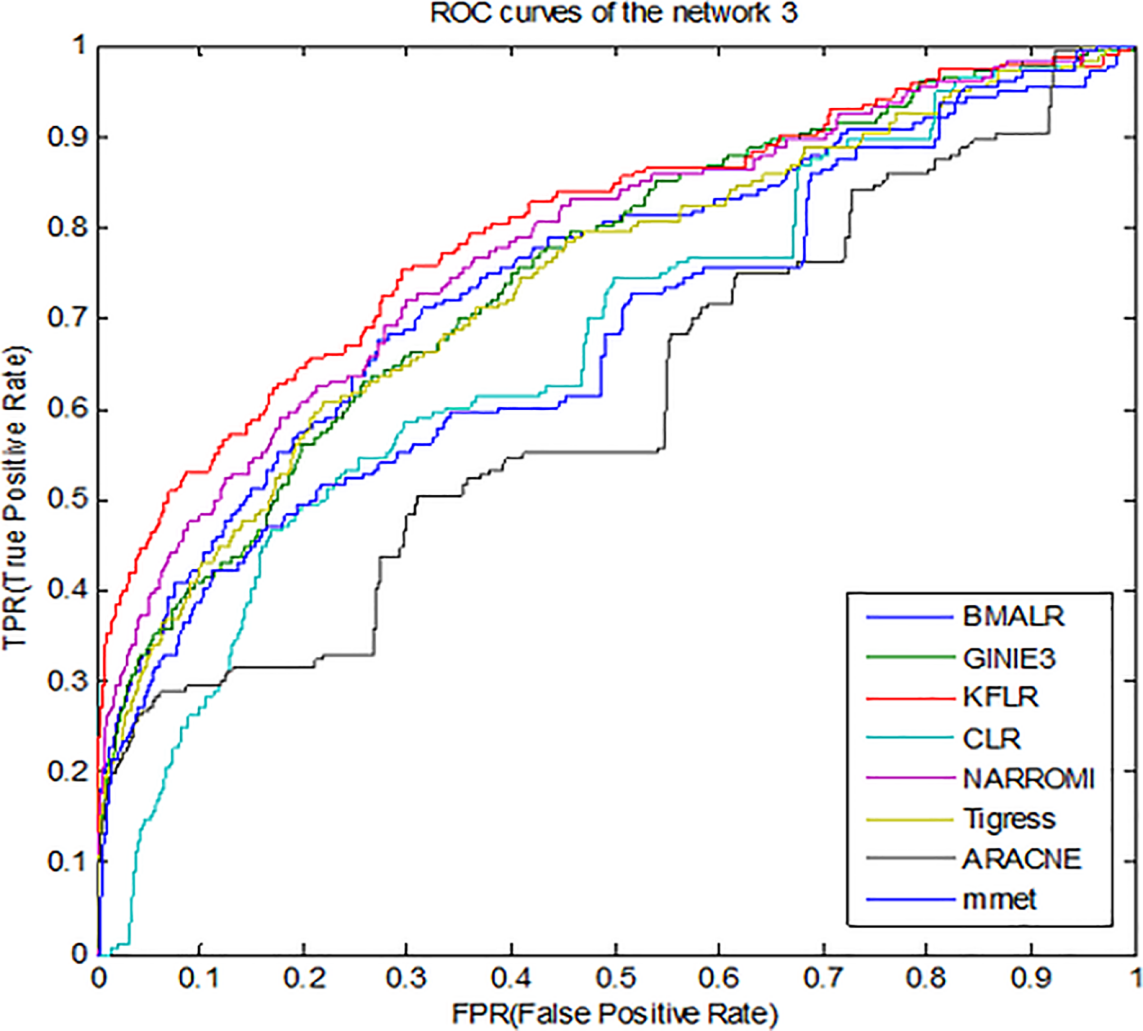
ROC curves for different methods in sub network3.

**Fig 6:**
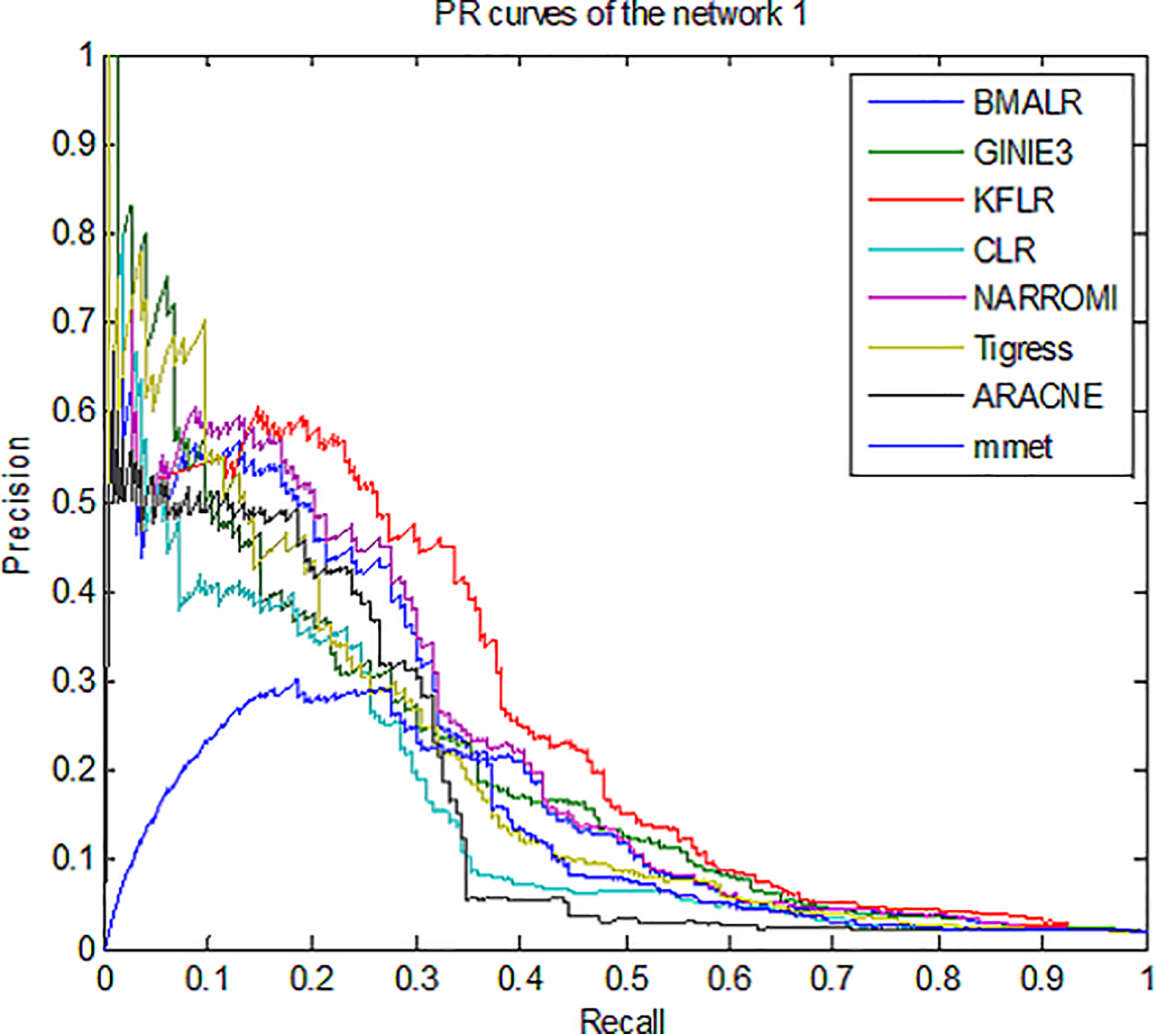
PR curves for different methods in sub network1.

**Fig 7:**
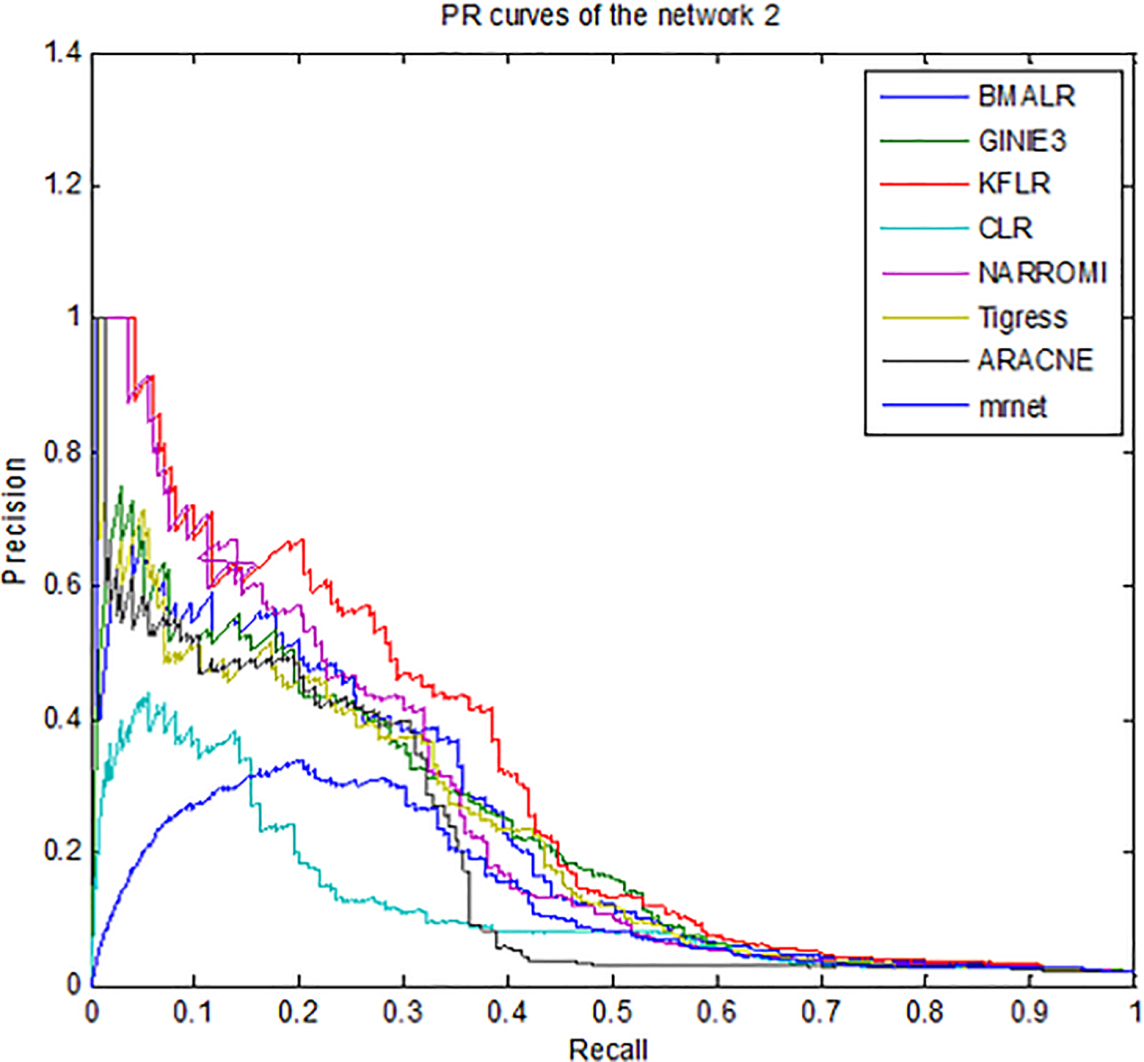
PR curves for different methods in sub network2.

**Fig 8:**
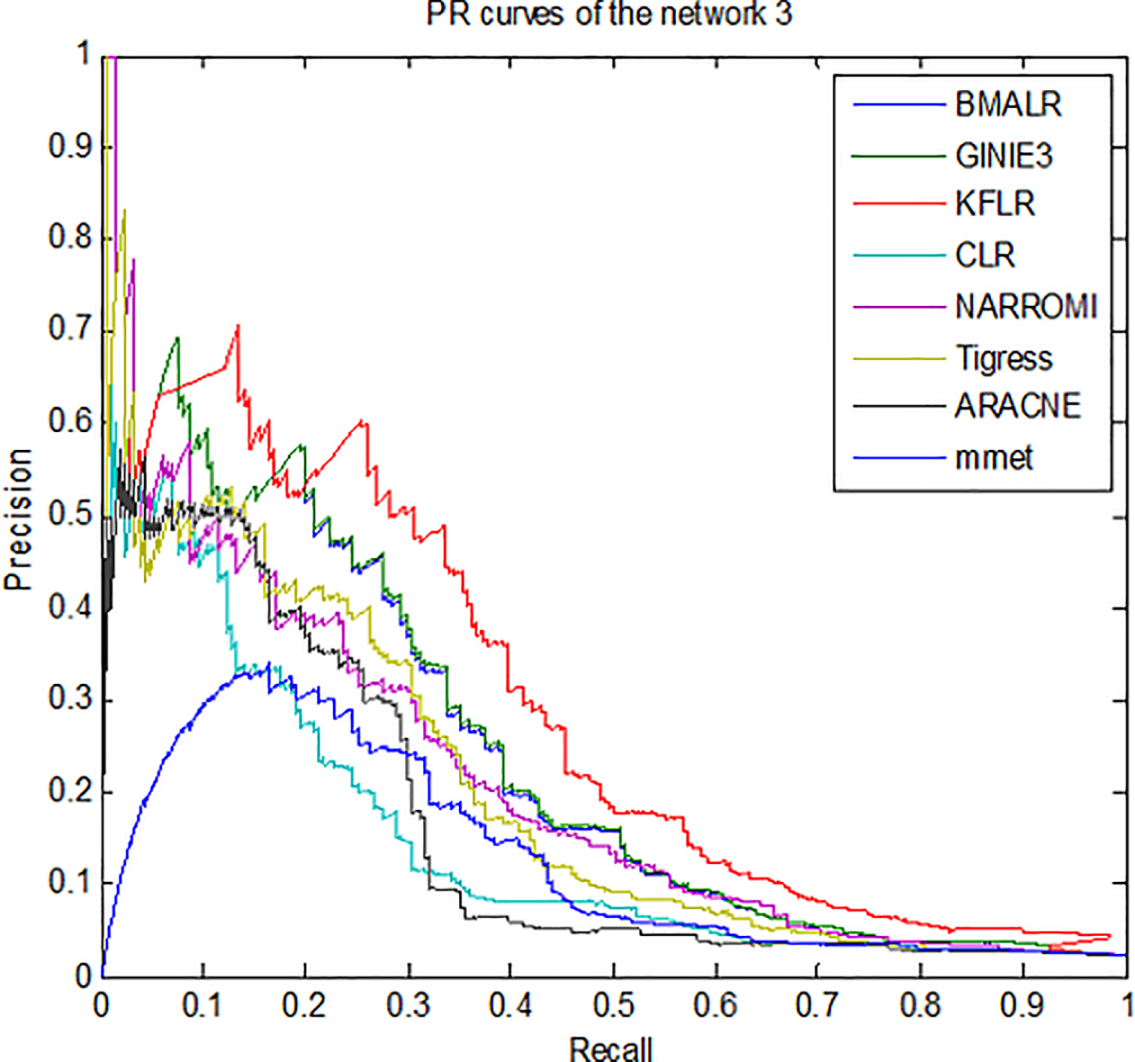
PR curves for different methods in sub network3.

Therefore, for better compression, *ROC* curves in noise data are drawn for three subnets and some methods is presented in Fig 3–5. According to all the figures, the *KFLR* method generally has a better result. Also, the *PR* curves in noise data are shown in Fig 6–8 for some methods and three subnets individually. According to all the *PR* figures, the *KFLR* approach in general has better and more accurate results.

### Performance comparison on the IRMA dataset

The different GRN inference methods were applied to reconstruct the *IRMA* (*In vivo* Reverse-engineering and Modeling Assessment) network. Table 5 shows the *AUPRs* of the GRNs inferenced in the noise data and in the main data. In the main data, *KFLR* is competitive with *BGRMI* method when inferring the network from the switch-on data. In the case of the switch-off data, *KFLR* had the highest accuracy. But in the noise data *KFLR* outperform other method. These results show that *KFLR* performs well on *in-silico* datasets and on *in-vivo* experimental data.

**Table 5:** AUPRs of the *In Vivo* IRMA network.

*Data*	*Without noise*		*With noise*	
*Method*	*switch-on Dataset*	*switch-off Dataset*	*switch-on Dataset*	*switch-off Dataset*
*BMALR*	0.634	0.336	0.586	0.308
*GINIE3*	0.62	0.347	0.543	0.289
*MRNET*	0.417	0.324	0.358	0.217
*ARACNE*	0.472	0.358	0.412	0.271
*BGRMI*	0.904	0.574	0.762	0.354
*CLR*	0.423	0.372	0.353	0.254
*G1DBN*	0.6	0.313	0.521	0.211
*NARROMI*	0.518	0.472	0.328	0.352
*TIGRESS*	0.714	0.452	0.592	0.376
*GENIRF*	0.672	0.327	0.581	0.312
*MIBNI*	0.656	0.348	0.582	0.354
*FBISC*	0.478	0.372	0.434	0.292
*CMI2NI*	0.721	0.456	0.589	0.371
*KFLR*	**0.896**	**0.721**	**0.834**	**0.709**

## Conclusion

In this paper, a new method was proposed to improve the accuracy of reconstructed GRN from time series gene expression data by using two approachs, i.e, the false-positive interactions deletion and the inference using model averaging. In this paper, by using *CMI* and *MI*, false-positive interactions were deleted and in the model averaging approach, Kalman filter was proposed to compute the posterior probabilities of the edges from possible regulators to the target gene with the combination of Bayesian model averaging and linear regression methods. The Kalman filter is a linear state-space model that operates recursively on noisy and time series input gene expression data to produce a statistically optimal estimate of the gene regulatory network. The results on the benchmark gene regulatory networks from the *DREAM4* challenge and in Vivo *IRMA* Network showed that the proposed method significantly outperforms other state-of-the-art methods. Also, it was established that this method is more robust to the noisy data.

## Supporting information

S1 Data Gene expression dataset of the DREAM4 and IRMA. (RAR)
